# Evidence for an Epistatic Effect between *TP53* R72P and *MDM2* T309G SNPs in HIV Infection: A Cross-Sectional Study in Women from South Brazil

**DOI:** 10.1371/journal.pone.0089489

**Published:** 2014-02-28

**Authors:** Fernando Pires Hartwig, Ludmila Gonçalves Entiauspe, Emily Montosa Nunes, Fernanda Martins Rodrigues, Tiago Collares, Fabiana Kömmling Seixas, Mariângela Freitas da Silveira

**Affiliations:** 1 Postgraduate Program in Epidemiology, Department of Social Medicine, Faculty of Medicine, Federal University of Pelotas, Pelotas, Rio Grande do Sul, Brazil; 2 Postgraduate Program in Biotechnology, Technology Development Center (Biotechnology Unit), Federal University of Pelotas, Pelotas, Rio Grande do Sul, Brazil; 3 Molecular and Cellular Oncology Research Group, Biotechnology Unit, Technology Development Center, Federal University of Pelotas, Pelotas, Rio Grande do Sul, Brazil; 4 Maternal and Child Department, Faculty of Medicine, Federal University of Pelotas, Pelotas, Rio Grande do Sul, Brazil; University of Saarland Medical School, Germany

## Abstract

**Objective:**

To investigate the associations of *TP53* R72P and *MDM2* T309G SNPs with HPV infection status, HPV oncogenic risk and HIV infection status.

**Design:**

Cross-sectional study combining two groups (150 HIV-negative and 100 HIV-positive) of women.

**Methods:**

Data was collected using a closed questionnaire. DNA was extracted from cervical samples. HPV infection status was determined by nested-PCR, and HPV oncogenic risk group by Sanger sequencing. Both SNPS were genotyped by PCR-RFLP. Crude and adjusted associations involving each exposure (R72P and T309G SNPs, as well as 13 models of epistasis) and each outcome (HPV status, HPV oncogenic risk group and HIV infection) were assessed using logistic regression.

**Results:**

R72P SNP was protectively associated with HPV status (overdominant model), as well as T309G SNP with HPV oncogenic risk (strongest in the overdominant model). No epistatic model was associated with HPV status, but a dominant (R72P over T309G) protective epistatic effect was observed for HPV oncogenic risk. HIV status was strongly associated (risk factor) with different epistatic models, especially in models based on a visual inspection of the results. Moreover, HIV status was evidenced to be an effect mediator of the associations involving HPV oncogenic risk.

**Conclusions:**

We found evidence for a role of R72P and T309G SNPs in HPV status and HPV oncogenic risk (respectively), and strong associations were found for an epistatic effect in HIV status. Prospective studies in larger samples are warranted to validate our findings, which point to a novel role of these SNPs in HIV infection.

## Introduction

Infection susceptibility variabilityamong individuals is observable in many infectious diseases. Understanding such variation and identifying its causal factors may have important implications for clinical practice and population health. Although intra and inter-population variation of viral infection susceptibility is attributable to several factors, host genetics likely plays an important role. Regarding HIV infection, a common example of genetic resistance is the *CCR5* 32-bp deletion. This allele, when transcribed and translated, results in a non-functional receptor, thus providing resistance to HIV infection [1]. Importantly, these findings are currently being incorporated into therapeutic approaches [2], [3], thus evidencing that understanding the genetic basis ofviral infection susceptibility have practical implications for human health. In this regard, several genome-wide association studies (GWAS) have been conducted to investigate the roles of host genetics inHIV load and/or disease progression [4]–[14], and, more recently, some GWAS focused on genetic factors associated with HIV acquisition in different populations [15]–[20]. Such attempts indicate the importance given to host genetics regarding HIV pathogenesis.

Considering that HIV infection is considered a major risk factor for HPV infection, the identification of host genetic factors involved with HIV infection may also have implications for HPV-related outcomes. Given the well-established roles of HPV in cancer, the majority of the studies involving HPV and host genetics are related with cancer development and/or progression associated with infection by oncogenic HPV strains. In this context, p53 pathway genes have beenextensively studied given that oncogenic HPV E6 orchestrates, in association with E6AP, p53 degradation by the ubiquitin-proteasome system[21]. Theoncogenic effects of HPVhavealso been investigatedepidemiologically (in different populations) using genetic variants in p53 pathway genes, including the *TP53* R72P SNP (rs1042522) [22]–[24], which was evidenced to interfere with p53 apoptotic and transcriptional functions[25]–[27]. More recently, the *MDM2* T309G SNP (rs2279744) [28]–[30], which influences p53 activity by affecting *MDM2* transcription, have also been investigated in similar contexts[31], [32].

In a recent review, different biological mechanisms by which interfering with p53 pathway may impact viral infection (or virus persistence after exposure to it) wereproposed [33]. In addition, there are substantial amounts of *in vitro* evidence supporting a functional relationship between p53 and different HIV proteins (with evidence for implicationsfor p53-mediated apoptosis), as well assome evidence regarding MDM2. These functional relationships with the p53 pathway have been evidenced for gp120 [34], [35], Rev, Tat [36] and Vpu[37] proteins. Considering the roles of R72P and T309G SNPs in p53 apoptosis and regulation (respectively) and the evidence for a role of p53 pathway in HPV and HIV biology, we aimed to investigate the association of these SNPs with HPV infection status, HPV oncogenic risk and HIV infection status.

## Materials and Methods

### Study Design and Participants

We performed a cross-sectional study, selecting two independent groups of women based on HIV status (determined by medical diagnosis). From May2010to May2011, 250 (150 HIV-negative and 100 HIV-positive)women seekinggynecologic careat the gynecological ambulatory clinic of Faculty of Medicine of Federal University of Pelotas (South Brazil) that fulfilledeligibility criteria (not pregnant, sexually active, and not menstruating) and agreed to participate were sequentially included in the study. Data were collected using an adapted version of a closed questionnaire [38], which was applied by a trained female interviewer. Routine gynecological exams (cervicitis indicators, visual inspection with acetic acid and Lugol's iodine), were performed and included in the questionnaire, as well the patient's recorded information (last Pap test result).

### Ethics Statement

The study was approved by the Ethics Committee of the Faculty of Medicine of Federal University of Pelotas (June 2009). Written informed consent was obtained from all participants. All procedures were performed in accordance with the Helsinki Declaration guidelines.

### DNA Collection

Cervical samples were collected with a cytobrushand placed into1.5 mlmicrotubescontaining300ml ofCell Lysis Solution (Puregene™ DNA Extraction Kit,*Gentra Systems* Minneapolis, MN). The material was enzymatically digested using 1.5 µl of proteinase K (10 mg/ml, New England Biolabs, MA) and incubatedovernightat room temperature. DNA was extracted according to manufacturer's specifications.

### HPV Detection and Genotyping

HPV detectionwas performed using nested-PCRintworounds: amplification of a 450 bp fragment using the MY09/11 primer pair [39] and amplification of a 140 bp fragment using the GP5/6 primer pair [40]. MY90/11 and GP5/6 PCRs (final reaction volume of 25 µl) were performed as follows: initial denaturation for 9 min at 95°C; 40 cycles of denaturation (for 1 min at 95°C and for 30 s at 94°C, respectively), primer annealing (for 1 min at 55°C and 30 s at 45°C, respectively), and extension (for 1 min and for 30 s, respectively, at 72°C); and final extension for 5 min at 72°C[41], [42]. PCR amplicons were visualized on 2.0% agarose gels stained with GelRed™ (Biotium Inc., CA). HPV-positive amplicons (from the second nested-PCR round) were purified using Gel Band purification kit (GE Healthcare, USA) according to manufacturer'sinstructions. To determine HPVoncogenic risk group (i.e., HPV genotype), Sanger sequencing was performed in a MegaBACE 1000 DNA sequencer (GE Healthcare, USA) using Dynamic ET-terminator technology. Chromatograms were assembled and analyzed using the ContigExpress module of the Vector NTI 10.0 suite (Invitrogen, USA). The assembled sequences were submitted to BLAST alignment (www.nci.nlm.gov/BLAST) against sequences available in GenBank.

Both SNPs were genotyped by PCR-RFLP using GoTaq qPCR Master Mix (Promega, USA) (in 12 µl reactions) with primers, restriction enzymes and PCR conditionsdescribed previously [43]–[45]. Briefly, for the R72P SNP, the 199 bpamplicon was cleaved using *Bst*UI (New England Biolabs, MA) and loaded on 2.5% agarose gel stained with GelRed™ (Biotium Inc., CA). Genotyping was performed as follows: one fragment of 199 bp corresponds to P72P genotype; three fragments of 199 bp, 113 bp and 86 bp correspond to R72P genotype; and two fragments of 113 bp and 86 bp correspond to R72R genotype. For the T309G SNP, the 157 bpamplicon was cleaved by *Msp*A1I (New England Biolabs, MA) and loaded on 2.5% agarose gel stained with GelRed™. Genotyping was performed as follows: one fragment of 157 bp corresponds to T309T genotype; three fragments of 157 bp, 109 bp and 48 bp correspond to T309G genotype; and two fragments of 109 bp and 48 bp correspond to G309G genotype.

### Statistical Analyses

Analyses were performed in R (version 3.0.1, http://www.r-project.org/). Descriptive analyses were stratified according to HIV infection status for both SNPs [including assessing Hardy-Weinberg equilibrium (HWE) by Fisher's exact test using the “genetics” R package: http://cran.r-project.org/web/packages/genetics/], status of HPV infection (positive or negative), HPV oncogenic risk (high or low) and potential confounding variables (i.e., skin color, achieved schooling in years, family income in minimum salaries and age). Skin color was a categorical variable defined by interviewer's observation. Achieved schooling in years was categorized in illiterate, 1–4, 5–8, 9–11 and 11 or more according to the formal educational system that the recruited women frequented [composed of: 8 years or Primary Education, which changes substantially after the first 4 years; Secondary Education, composed of 3 years (in a total of 11 years); and Higher Education]. Age was categorized in groups of (approximately) 5 years (adjusting the age limits of some groups to avoid categories with very few individuals) for a more detailed comparison between HIV groups. Crude comparisons between HIV strata were performed by either chi-squared or Fisher's exact test (using the “gmodels” R package: http://cran.r-project.org/web/packages/gmodels/index.html).

Importantly, any confounding effect of age, education and family income (after adjusting for skin color) on the association between a genetic factor and a given trait is expected to occur by chance (since an individual's genotype is not influenced by environmental factors), thus imposing a difficulty to establish an adequate conceptual framework to select confounding variables. Therefore, we used a statistical-oriented approach, although skin color was invariable adjusted for. The reason for doing so is the well-known possibility of confounding in SNP-outcome analyses due to population stratification. Since both socioeconomic status (in several populations including Brazil) and genotypic frequencies of the vast majority of SNPs vary substantially according to skin color, the last can create spurious associations between genetic factors and outcomes influenced by socioeconomic factors. This is relevant for this manuscript since the last are known to have profound implications for sexually transmitted diseases.

The confounding variable selection was performed by stepwise backwards selection (critical P = 0.2 according to likelihood-ratio chi-squared test using the “car” R package: http://cran.r-project.org/web/packages/car/index.html). Since skin color was invariably adjusted for due to conceptual considerations, it was not subjected to removal. This process was performed having, as the independent variable, each outcome of the study (i.e., HPV status, HPV oncogenic risk and HIV status) and each main exposure (R72P and T309G genotypes, as well as the genotypes combined). These analyses were performed by logistic and multinomial logistic regression (using the “nnet” R package: http://cran.r-project.org/web/packages/nnet/index.html), respectively. The variables that remained in the final models of theoutcome and the genetic exposure of interest were considered confounding factors for the associations involving such exposure-outcome pair.

Associations involving R72P and T309G SNPs and study outcomes were assessed in crude and adjusted logistic regression [estimating odds ratio (OR)] models. Five genetic models (obtained using the “SNPassoc” R package: http://cran.r-project.org/web/packages/SNPassoc/index.html) were tested: codominant or genotypic (i.e., each genotype is coded as a distinct category), additive (i.e., the SNPs are coded numerically according to the number of variant alleles), overdominant (i.e., homozygous genotypes  = 0 and heterozygous genotype  = 1), dominant (i.e., homozygous wild  = 0; heterozygous and homozygous variant genotype  = 1) and recessive (i.e., homozygous wild and heterozygous genotypes  = 0 and homozygous variant genotype  = 1), withthe category corresponding to (or containing) the wild-type homozygous genotype as the reference group (as described elsewhere [46]).

For the analyses of epistasis, a total of 13 epistaticmodels were tested using crude and adjusted logistic regression models. Eleven of them were described elsewhere [46]. Briefly, the following epistatic models were tested: dominant epistasis [both R72P over T309G (reference: R72R T309T; category 1: R72R G309_; category 2: P72_ _309_) and vice-versa (reference: R72R T309T; category 1: P72_ T309T; category 2: _72_ G309_)], recessive epistasis [both R72P over T309G (reference: R72_ T309_; category 1: R72_ G309G; category 2: P72P _309) and vice-versa (reference: R72_ T309_; category 1: R72R T309_; category 2: _72_ G309G)], dominant and recessive epistasis [both R72P over T309G (reference: remaining genotypic combinations; category 1: P72_ G309G) and vice-versa (reference: remaining genotypic combinations; category 1: P72P G309_)], double dominant epistasis without cumulative effect (reference: R72R T309T; category 1: remaining genotypic combinations), double recessive epistasis without cumulative effect (reference: R72_ T309_; category 1: remaining genotypic combinations), double dominant epistasis with cumulative effect (reference: R72R T309T; category 1: remaining genotypic combinations; category 2: P72_ G309_), double recessive epistasis with cumulative effect (reference: R72_ T309_; category 1: remaining genotypic combinations; category 2: P72P G309G) and quantitative (number of P72 and G309 alleles). After testing theseepistatic models, two additional ones were elaborated based on an exploratory visual inspection (plot obtained using the “plotrix” R package: http://cran.r-project.org/web/packages/plotrix/index.html) of the OR [and associated 95% confidence intervals (95% CI)] resulting from an adjusted logistic regression model of HIV status (dependent variable) on combined genotypes of R72P and T309G SNPs (independent variable). This visual method was described elsewhere [46] and is aimed at identifying patterns that may indicate anepistatic relationship not reflected in the other models. For the association analyses, P<0.05 was considered statistically significant and P<0.10 (but ≥0.05) was considered of marginal significance.

Given our limited sample size and the practical/logistic impossibility of increasing it, power analyses were performed to estimate the statistical power of this study for different OR values. These analyses were performed by simulations (see Methods S1 for details).

## Results

### Sample Description

The characteristics of the sample (stratified according to HIV infection status) are shown in table 1. There were statistically significant differences between HIV-positive and HIV-negative regarding the two HPV-related outcomes, age, schooling (P<0.001) and skin color (P = 0.005). Family income did not significantly differ between HIV strata (P = 0.389). In this initial analysis, no significant differenceswere observed between HIV strata regarding the genotypic frequencies of R72P (P = 0.200) and T309G (P = 0.543) SNPs. Moreover, there was no evidence for departures from HWE regarding R72P and T309G SNPs in the total sample (P>0.999 and P = 0.102, respectively) and within skin color strata (white: P = 0.642 and P = 0.474; black: P>0.999 and P>0.999; brown: P = 0.797 and P = 0.331).

**Table 1:** Descriptive analyses of the sample, comparing the HIV groups.

Variable	Categories	HIV status		P-value
		Negative (n = 250)	Positive (n = 100)	
**HPV status**	Positive	176 (70.4%)	32 (32.0%)	<0.001*
	Negative	74 (29.6%)	68 (68.0%)	
**HPV oncogenic risk**	Low-risk	7 (10.1%)	38 (66.7%)	<0.001*
	High-risk	62 (89.9%)	19 (33.3%)	
**Age groups**	18–24	43 (17.2%)	3 (3.0%)	<0.001†
	25–30	55 (22.0%)	10 (10.0%)	
	31–35	45 (18.0%)	17 (17.0%)	
	36–39	50 (20.0%)	17 (17.0%)	
	40–45	57 (22.8%)	53 (53.0%)	
**Skin color**	White	164 (65.6%)	51 (51.0%)	0.005*
	Black	31 (12.4%)	26 (26.0%)	
	Brown	55 (22.0%)	23 (23.0%)	
**Family income**	≤1: 50	50 (26.6%)	27 (30.7%)	0.389*
**(minimum salaries)**	>1, ≤2	80 (42.6%)	33 (37.5%)	
	>2, ≤3	46 (24.5%)	18 (20.5%)	
	≥4	12 (6.4%)	10 (11.4%)	
**Achieved schooling**	Illiterate	4 (1.6%)	0 (0.0%)	<0.001*
**(years)**	1–4	32 (13.0%)	22 (23.2%)	
	5–8	93 (37.8%)	43 (45.3%)	
	9-10	36 (14.6%)	0 (0.0%)	
	≥11	81 (32.9%)	30 (31.6%)	
**R72P**	R72R	27 (10.8%)	18 (18.0%)	0.200*
	R72P	118 (47.2%)	43 (43.0%)	
	P72P	105 (42.0%)	39 (39.0%)	
**T309G**	T309T	35 (14.0%)	16 (16.0%)	0.543*
	T309G	101 (40.4%)	45 (45.0%)	
	G309G	114 (45.6%)	39 (39.0%)	

^*^Fisher's exact test.

† Chi-square test.

The observed differences are in the expected directionregardingHPV infection prevalence (70.4% in HIV-positive and 32.0% in HIV-negative women), age (prevalence of age groups: 18–24, 17.2% and 3.0%; 25–30, 22.0% and 10.0%; 31–35, 18.0% and 17.0%; 36–39, 20.0% and 17.0%; and 40–45, 22.8% and 53% in HIV-positive and HIV-negative groups, respectively), skin color (prevalence of skin color groups: white, 65.6% and 51.0%; black, 12.4% and 26.0%; and brown,22.0% and 23.0% in HIV-positive and HIV-negative women, respectively) and achieved schooling in years (prevalence of schooling categories: illiterate, 1.6% and 0.0%; 1–4, 13.0% and 23.2%; 5–8, 37.8% and 45.3%; 9–10, 14.6% and 0.0%; and 11 or more, 32.9% and 31.6%, in HIV-positive and HIV-negative women, respectively). Although the genotypic frequencies of none of the SNPs significantly differed between HIV strata, the heterogeneity of these groups was considered relevant for the association analyses.

### Associations of R72P and T309G SNPs with HPV Outcomes and HIV Status

For R72P SNP, only skin color was considered a covariate for all outcomes. For T309G SNP, in addition to skin color, age was also considered a covariate for HPV status and HIV status (tables S1 and S2). Crude and adjusted analyses for the associations of R72P and T309G SNPs with the outcomes are shown in tables 2–4. For HPV status, no significant associations with T309G SNP were observed (P≥0.500). Regarding R72P SNP, a marginal association was observedfor the codominant model [crude (genotypes R72P and P72P, respectively): OR (95% CI), 0.63 (0.40–1.00) and 1.16 (0.59–2.28); P = 0.071; adjusted: OR (95% CI), 0.62 (0.39–0.99) and 1.14 (0.57–2.28); P = 0.065] and there was a significant association for the overdominant model [crude: OR (95% CI), 0.61 (0.39–0.94); P = 0.024; adjusted: OR (95% CI), 0.60 (0.39–0.93); P = 0.021]. Indeed, the OR patterns observed for the codominant model indicatea protective overdominant effect.

**Table 2:** Crude and adjusted associations [showing OR (95% CI) and p-values] between R72P and T309G SNPs and HPV status.

Genetic	R72P		T309G	
model*	Crude	Adjusted	Crude	Adjusted
**Codominant**	P = 0.071	P = 0.065	P = 0.786	P = 0.859
A/A	1 (Reference)	1 (Reference)	1 (Reference)	1 (Reference)
A/a	0.63 (0.40–1.00)	0.62 (0.39–0.99)	1.18 (0.74–1.87)	1.14 (0.71–1.84)
a/a	1.16 (0.59–2.28)	1.14 (0.57–2.28)	1.12 (0.58–2.12)	1.08 (0.54–2.11)
**Overdominant**	P = 0.024	P = 0.021	P = 0.542	P = 0.612
A/A-a/a	1 (Reference)	1 (Reference)	1 (Reference)	1 (Reference)
A/a	0.61 (0.39–0.94)	0.60 (0.39–0.93)	1.14 (0.74–1.76)	1.12 (0.72–1.74)
**Additive**	P = 0.649	P = 0.602	P = 0.602	P = 0.707
N°-of “a” alleles	0.93 (0.68–1.27)	0.92 (0.66–1.27)	1.08 (0.80–1.47)	1.06 (0.77–1.46)
**Dominant**	P = 0.146	P = 0.125	P = 0.500	P = 0.601
A/A	1 (Reference)	1 (Reference)	1 (Reference)	1 (Reference)
A/a-a/a	0.73 (0.47–1.12)	0.71 (0.46–1.10)	1.16 (0.75–1.79)	1.13 (0.72–1.77)
**Recessive**	P = 0.227	P = 0.229	P = 0.924	P = 0.991
A/A-A/a	1 (Reference)	1 (Reference)	1 (Reference)	1 (Reference)
a/a	1.47 (0.78–2.77)	1.48 (0.78–2.82)	1.03 (0.56–1.87)	1.00 (0.53–1.87)

^*^“A” and “a” correspond to wild-type (i.e., either R72 or T309) and variant alleles (i.e., either P72 or G309), respectively.

**Table 3:** Crude and adjusted associations [showing OR (95% CI) and p-values] between R72P and T309G SNPs and HPV oncogenic risk.

Genetic	R72P		T309G	
model*	Crude	Adjusted	Crude	Adjusted
**Codominant**	P = 0.680	P = 0.553	P = 0.024	P = 0.019
A/A	1 (Reference)	1 (Reference)	1 (Reference)	1 (Reference)
A/a	1.43 (0.65–3.21)	1.54 (0.69–3.55)	0.35 (0.15–0.78)	0.32 (0.13–0.74)
a/a	1.19 (0.40–3.85)	1.48 (0.46–5.22)	0.91 (0.28–3.27)	0.81 (0.24–2.99)
**Overdominant**	P = 0.410	P = 0.381	P = 0.006	P = 0.005
A/A-a/a	1 (Reference)	1 (Reference)	1 (Reference)	1 (Reference)
A/a	1.37 (0.65–2.99)	1.41 (0.66–3.09)	0.36 (0.17–0.75)	0.34 (0.16–0.73)
**Additive**	P = 0.545	P = 0.350	P = 0.321	P = 0.244
N°-of “a” alleles	1.17 (0.70–2.01)	1.30 (0.75–2.32)	0.77 (0.45–1.30)	0.73 (0.42–1.24)
**Dominant**	P = 0.410	P = 0.277	P = 0.033	P = 0.021
A/A	1 (Reference)	1 (Reference)	1 (Reference)	1 (Reference)
A/a-a/a	1.36 (0.65–2.84)	1.53 (0.71–3.33)	0.43 (0.19–0.94)	0.40 (0.17–0.87)
**Recessive**	P = 0.969	P = 0.761	P = 0.344	P = 0.394
A/A-A/a	1 (Reference)	1 (Reference)	1 (Reference)	1 (Reference)
a/a	1.02 (0.36–3.16)	1.19 (0.40–3.90)	1.67 (0.59–5.49)	1.60 (0.56–5.29)

^*^“A” and “a” correspond to wild-type (i.e., either R72 or T309) and variant alleles (i.e., either P72 or G309), respectively.

**Table 4:** Crude and adjusted associations [showing OR (95% CI) and p-values] between R72P and T309G SNPs and HIV status.

Genetic	R72P		T309G	
model*	Crude	Adjusted	Crude	Adjusted
**Codominant**	P = 0.209	P = 0.432	P = 0.528	P = 0.168
A/A	1 (Reference)	1 (Reference)	1 (Reference)	1 (Reference)
A/a	0.98 (0.59–1.63)	0.90 (0.54–1.52)	1.30 (0.79–2.17)	1.60 (0.91–2.84)
a/a	1.79 (0.88–3.61)	1.45 (0.69–2.99)	1.34 (0.66–2.65)	1.85 (0.83–4.07)
**Overdominant**	P = 0.476	P = 0.405	P = 0.431	P = 0.254
A/A-a/a	1 (Reference)	1 (Reference)	1 (Reference)	1 (Reference)
A/a	0.84 (0.53–1.34)	0.82 (0.51–1.31)	1.21 (0.75–1.93)	1.35 (0.81–2.25)
**Additive**	P = 0.205	P = 0.517	P = 0.305	P = 0.073
N°-of “a” alleles	1.25 (0.89–1.75)	1.12 (0.79–1.60)	1.19 (0.86–1.64)	1.41 (0.97–2.05)
**Dominant**	P = 0.606	P = 0.984	P = 0.259	P = 0.064
A/A	1 (Reference)	1 (Reference)	1 (Reference)	1 (Reference)
A/a-a/a	1.13 (0.71–1.83)	1.00 (0.62–1.64)	1.31 (0.82–2.11)	1.65 (0.97–2.86)
**Recessive**	P = 0.077	P = 0.216	P = 0.634	P = 0.347
A/A-A/a	1 (Reference)	1 (Reference)	1 (Reference)	1 (Reference)
a/a	1.81 (0.94–3.45)	1.53 (0.77–2.97)	1.17 (0.60–2.19)	1.42 (0.68–2.87)

^*^“A” and “a” correspond to wild-type (i.e., either R72 or T309) and variant alleles (i.e., either P72 or G309), respectively.

For HPV oncogenic risk, the results were somehow opposite. No significant associations were observed for R72P SNP (P≥0.277). Regarding T309G SNP, significant associations were observed for codominant[crude (genotypes T309G and G309G, respectively): OR (95% CI), 0.35 (0.15–0.78) and 0.91 (0.28–3.27); P = 0.024; adjusted: OR (95% CI), 0.32 (0.13–0.74) and 0.81 (0.24–2.99); P = 0.019], overdominant[crude: OR (95% CI), 0.36 (0.17–0.75); P = 0.006; adjusted: OR (95% CI), 0.34 (0.16–0.73); P = 0.005] and dominant [crude: OR (95% CI), 0.43 (0.19–0.94); P = 0.033; adjusted; OR (95% CI), 0.40 (0.17–0.87); P = 0.021] models. Again, the codominant model already indicateda protective overdominant effect.

In contrast to HPV outcomes, only marginal associations were observed for HIV status. There were neither significant nor marginalassociations for R72P SNP (P≥0.205). Regarding T309G SNP, marginal associations were observed for additive [crude: OR (95% CI), 1.19 (0.86–1.64); P = 0.305; adjusted: OR (95% CI), 1.41 (0.97–2.05); P = 0.073] and dominant [crude: OR (95% CI), 1.31 (0.82–2.11); P = 0.259; adjusted: OR (95% CI), 1.65 (0.97–2.86); P = 0.064] models. Although marginal, these associations lead to the speculation that HIV status could be an (at least partial) effect mediator of the associations between T309GSNP and HPV oncogenic risk. In this regard, the analysis of association between this SNP and HPV oncogenic risk were repeated with the inclusion of HIV status as a covariate (table S3). By doing so, only the overdominant model remained, although attenuated, statistically significant [OR (95% CI), 0.38 (0.14–0.96); P = 0.040]. The same was performed for the analysis of association between R72P SNP and HPV status, but no substantial differences were observed [overdominant model: OR (95% CI), 0.60 (0.38–0.96); P = 0.032], further evidencing an effect mediation role in HIV status in the associations between T309G SNP and HPV oncogenic risk.

### Epistatic Models Were Associated with HPV Oncogenic Risk and HIV Status

In addition to skin color, age was considered a covariate for HPV status and HIV status (table S4). Crude and adjusted analyses for the associations ofepistatic models and studyoutcomes are shown in tables 5–7. Noassociations were observed for HPV status (P≥0.192 and P≥0.129, respectively). RegardingHPV oncogenic risk, a significant association was observed for dominant epistasis with R72P SNP overcoming the effects of T309G SNP [model 1.1 (genotypic combinations R72P G309_ and R72_ _309_, respectively); crude: OR (95% CI), 0.24 (0.06–0.77) and 0.49 (0.13–1.53); P = 0.038; adjusted: OR (95% CI), 0.22 (0.06–0.73) and 0.54 (0.14–1.72); P = 0.024]. In addition, marginal associations were observed for dominant epistasis with T309G SNP overcoming the effects of R72P SNP [model 1.2 (genotypic combinations P72 T309T and _72_ G309_, respectively); crude: OR (95% CI), 0.59 (0.14–2.22) and 0.31 (0.08–0.94); P = 0.077; adjusted: OR (95% CI), 0.67 (0.15–2.63) and 0.31 (0.08–0.94); P = 0.060], dominant and recessive epistasis with R72P having a dominant effect given that G309G occurs[model 3.1; crude: OR (95% CI), 4.82 (0.84–90–98); P = 0.082; adjusted: OR (95% CI), 4.75 (0.83–89.77); P = 0.086] and double dominant epistasis without cumulative effect [model 4; crude: OR (95% CI), 0.37 (0.10–1.08); P = 0.069; adjusted: OR (95% CI), 0.37 (0.10–1.10); P = 0.074]. For the other models, no associations were observed (P≥0.159).

**Table 5:** Associations between 11 epistatic models and HPV status.

Epistaticmodel*	Genotypic	Crude		Adjusted	
	combination	OR (95% CI)	P-value	OR (95% CI)	P-value
Dominant (1.1)	R72R T309T	1 (Reference)	0.192	1 (Reference)	0.129
	R72R G309_†	1.45 (0.74–2.87)		1.44 (0.72–2.88)	
	P72_ _309_	0.91 (0.50–1.67)		0.83 (0.45–1.55)	
Dominant (1.2)	R72R T309T	1 (Reference)	0.778	1 (Reference)	0.815
	P72_ T309T	0.93 (0.48–1.82)		0.88 (0.44–1.76)	
	_72_ G309_	1.11 (0.61–2.03)		1.04 (0.57–1.93)	
Recessive (2.1)	R72_ T309_	1 (Reference)	0.479	1 (Reference)	0.378
	R72_ G309G	1.03 (0.53–1.96)		0.96 (0.49–1.88)	
	P72P _309_	1.48 (0.78–2.80)		1.58 (0.82–3.07)	
Recessive(2.2)	R72_ T309_	1 (Reference)	0.533	1 (Reference)	0.494
	R72R T309_	1.47 (0.74–2.90)		1.53 (0.76–3.07)	
	_72_ G309G	1.08 (0.58–1.99)		1.05 (0.55–1.97)	
Dominantand	Other¥	1 (Reference)	0.482	1 (Reference)	0.479
recessive (3.1)	P72_ G309G	0.75 (0.33–1.64)		0.75 (0.32–1.66)	
Dominantand	Other	1 (Reference)	0.952	1 (Reference)	0.888
recessive (3.2)	P72PG309_	0.97 (0.41–2.21)		1.06 (0.44–2.48)	
Double dominant(no	R72R T309T	1 (Reference)	0.876	1 (Reference)	0.966
effect accumulation) (4)	Other	1.05 (0.59–1.88)		0.99 (0.55–1.79)	
Double recessive (no	R72_ T309_	1 (Reference)	0.387	1 (Reference)	0.395
effect accumulation) (5)	Other	1.24 (0.76–2.01)		1.24 (0.75–2.03)	
Double dominant(with	R72R T309T	1 (Reference)	0.574	1 (Reference)	0.433
effect accumulation) (6)	Other	1.15 (0.63–2.12)		1.11 (0.61–2.08)	
	P72_ G309_	0.89 (0.47–1.72)		0.80 (0.41–1.57)	
Double recessive (with	R72_ T309_	1 (Reference)	0.661	1 (Reference)	0.602
effect accumulation) (7)	Other	1.22 (0.74–2.00)		1.20 (0.72–2.00)	
	P72P G309G	1.55 (0.28–8.51)		1.92 (0.34–11.01)	
Quantitative (8)	N° of P72 and	1.01 (0.81–1.26)	0.950	0.98 (0.78–1.24)	0.893
	G309 alleles				

^*^The epistatic models were numbered as described previously [46].

† The “_” indicates that the effect is irrespective of the allele. E.g., R72P G309_ represents the genotypic combinations R72P T309G - R72P G309G.

¥ Other: **3.1**:_72_ T309_ - R72R _309_. **3.2**: R72_ _309_ - _72_ T309T. **4**: P72_ _309_ - _72_ G309_. **5**: P72P _309_ - _72_ G309G. **6**: R72R G309_ - P72_ T309T. **7**: P72P T309_ - R72_ G309G.

**Table 6:** Associations between 11 epistatic models and HPV oncogenic risk.

Epistaticmodel*	Genotypic	Crude		Adjusted	
	Combination	OR (95% CI)	P-value	OR (95% CI)	P-value
Dominant (1.1)	R72R T309T	1 (Reference)	0.038	1 (Reference)	0.024
	R72P G309_†	0.24 (0.06–0.77)		0.22 (0.06–0.73)	
	P72_ _309_	0.49 (0.13–1.53)		0.54 (0.14–1.72)	
Dominant (1.2)	R72R T309T	1 (Reference)	0.077	1 (Reference)	0.060
	P72_ T309T	0.59 (0.14–2.22)		0.67 (0.15–2.63)	
	_72_ G309_	0.31 (0.08–0.94)		0.31 (0.08–0.94)	
Recessive (2.1)	R72_ T309_	1 (Reference)	0.608	1 (Reference)	0.649
	R72_ G309G	1.81 (0.58–6.87)		1.70 (0.54–6.50)	
	P72P _309_	1.11 (0.39–3.46)		1.25 (0.42–4.11)	
Recessive(2.2)	R72_ T309_	1 (Reference)	0.633	1 (Reference)	0.656
	R72R T309_	1.09 (0.35–3.78)		1.24 (0.38–4.53)	
	_72_ G309G	1.69 (0.59–5.59)		1.63 (0.57–5.43)	
Dominantand	Other¥	1 (Reference)	0.082	1 (Reference)	0.086
recessive (3.1)	P72_ G309G	4.82 (0.84–90.98)		4.75 (0.83–89.77)	
Dominantand	Other	1 (Reference)	0.876	1 (Reference)	0.768
recessive (3.2)	P72PG309_	1.12 (0.28–5.52)		1.24 (0.30–6.27)	
Double dominant(no	R72R T309T	1 (Reference)	0.069	1 (Reference)	0.074
effect accumulation) (4)	Other	0.37 (0.10–1.08)		0.37 (0.10–1.10)	
Double recessive (no	R72_ T309_	1 (Reference)	0.446	1 (Reference)	0.396
effect accumulation) (5)	Other	1.39 (0.60–3.37)		1.45 (0.62–3.54)	
Double dominant(with	R72R T309T	1 (Reference)	0.159	1 (Reference)	0.165
effect accumulation) (6)	Other	0.34 (0.09–1.02)		0.34 (0.09–1.04)	
	P72_ G309_	0.43 (0.11–1.47)		0.44 (0.11–1.52)	
Double recessive (with	R72_ T309_	1 (Reference)	0.743	1 (Reference)	0.697
effect accumulation) (7)	Other	1.41 (0.59–3.55)		1.45 (0.61–3.70)	
	P72P G309G	1.21 (0.11–26.53)		1.36 (0.12–30.58)	
Quantitative (8)	N° of P72 and	0.95 (0.65–1.39)	0.781	0.96 (0.66–1.42)	0.854
	G309 alleles				

^*^The epistatic models were numbered as described previously [46].

† The “_” indicates that the effect is irrespective of the allele. E.g., R72P G309_ represents the genotypic combinations R72P T309G - R72P G309G.

¥ Other: **3.1**: _72_ T309_ - R72R _309_. **3.2**: R72_ _309_ - _72_ T309T. **4**: P72_ _309_ - _72_ G309_. **5**: P72P _309_ - _72_ G309G. **6**: R72R G309_ - P72_ T309T. **7**: P72P T309_ - R72_ G309G.

**Table 7:** Associations between 11 epistatic models and HIV status.

Epistaticmodel*	Genotypic	Crude		Adjusted	
	combination	OR (95% CI)	P-value	OR (95% CI)	P-value
Dominant (1.1)	R72R T309T	1 (Reference)	0.009	1 (Reference)	0.004
	R72P G309_†	3.52 (1.54–8.88)		4.34 (1.79–11.57)	
	P72_ _309_	2.63 (1.23–6.29)		2.45 (1.09–6.10)	
Dominant (1.2)	R72R T309T	1 (Reference)	0.015	1 (Reference)	0.020
	P72_ T309T	3.03 (1.33–7.59)		2.57 (1.06–6.78)	
	_72_ G309_	2.80 (1.32–6.71)		3.09 (1.38–7.72)	
Recessive (2.1)	R72_ T309_	1 (Reference)	0.166	1 (Reference)	0.139
	R72_ G309G	1.28 (0.62–2.50)		1.46 (0.67–3.10)	
	P72P _309_	1.88 (0.96–3.61)		2.01 (0.95–4.20)	
Recessive(2.2)	R72_ T309_	1 (Reference)	0.156	1 (Reference)	0.151
	R72R T309_	1.96 (0.97–3.92)		1.97 (0.90–4.25)	
	_72_ G309G	1.29 (0.66–2.45)		1.53 (0.73–3.14)	
Dominantand	Other¥	1 (Reference)	0.140	1 (Reference)	0.420
recessive (3.1)	P72_ G309G	0.50 (0.16–1.24)		0.65 (0.20–1.79)	
Dominantand	Other	1 (Reference)	0.948	1 (Reference)	0.903
recessive (3.2)	P72PG309_	0.97 (0.37–2.31)		1.06 (0.38–2.71)	0.903
Double dominant(no	R72R T309T	1 (Reference)	0.004	1 (Reference)	0.006
effect accumulation) (4)	Other	2.88 (1.38–6.78)		2.90 (1.33–7.08)	
Double recessive (no	R72_ T309_	1 (Reference)	0.093	1 (Reference)	0.060
effect accumulation) (5)	Other	1.56 (0.93–2.59)		1.72 (0.98–3.02)	
Double dominant(with	R72R T309T	1 (Reference)	0.007	1 (Reference)	0.011
effect accumulation) (6)	Other	3.26 (1.52–7.81)		3.29 (1.47–8.20)	
	P72_ G309_	2.31 (1.02–5.78)		2.32 (0.97–6.04)	
Double recessive (with	R72_ T309_	1 (Reference)	0.243	1 (Reference)	0.164
effect accumulation) (7)	Other	1.57 (0.92–2.64)		1.69 (0.95–3.00)	
	P72P G309G	1.41 (0.19–7.40)		2.29 (0.25–15.36)	
Quantitative (8)	N° of P72 and	1.23 (0.96–1.56)	0.098	1.28 (0.98–1.67)	0.074
	G309 alleles				

^*^The epistatic models were numbered as described previously [46].

† The “_” indicates that the effect is irrespective of the allele. E.g., R72P G309_ represents the genotypic combinations R72P T309G - R72P G309G.

¥ Other: **3.1**: _72_ T309_ - R72R _309_. **3.2**: R72_ _309_ - _72_ T309T. **4**: P72_ _309_ - _72_ G309_. **5**: P72P _309_ - _72_ G309G. **6**: R72R G309_ - P72_ T309T. **7**: P72P T309_ - R72_ G309G.

The most significant associations involving models of epistasis were observed for HIV status. The strongest ones were observed for models of dominant epistasis with R72P SNP overcoming the effects of T309G SNP [model 1.1 (genotypic combinations R72P G309_ and R72_ _309_, respectively);crude: OR (95% CI), 3.52 (1.54–8.88) and 2.63 (1.23–6.29); P = 0.009; adjusted: OR (95% CI), 4.34 (1.79–11.57) and 2.45 (1.09–6.10); P = 0.004] and double dominant epistasis without cumulative effects (model 4; crude: OR (95% CI), 2.88 (1.38–6.78); P = 0.004; adjusted: OR (95% CI), 2.90 (1.33–7.08); P = 0.006]. There were significant associations also for dominant epistasis with T309G SNP overcoming the effects of R72P SNP [model 1.2 (genotypic combinations P72 T309T and _72_ G309_, respectively); crude: OR (95% CI), 3.03 (1.33–7.59) and 2.80 (1.32–6.71); P = 0.015; adjusted: OR (95% CI), 2.57 (1.06–6.78) and 3.09 (1.38–7.72); P = 0.020] and double dominant epistasis with cumulative effect [model 7 (genotypic combinations P72P T309_ - R72_ G309G and P72P G309G, respectively); crude: OR (95% CI), 3.26 (1.52–7.81) and 2.31 (1.02–5.78); P = 0.007; adjusted: OR (95% CI), 3.29 (1.47–8.20) and 2.32 (0.97–6.04); P = 0.011]. Marginal associations were observed for double recessive epistasis without cumulative effects [model 5; crude: OR (95% CI), 1.56 (0.93–2.59); P = 0.093; adjusted: OR (95% CI), 1.72 (0.98–3.02); P = 0.060] and quantitative [model 8; crude: OR (95% CI), 1.23 (0.96–1.56); P = 0.098; adjusted: OR (95% CI), 1.28 (0.98–1.67); P = 0.074] models. Since statistically significant associations of different epistaticmodels withHPV oncogenic risk and HIV status were observed, the adjusted analyses for the first were repeated, including HIV status as a covariate (table S5). The significant associations were not maintained (P≥0.142), thus suggestinga mediation effectof HIV status.

**Figure 1:**
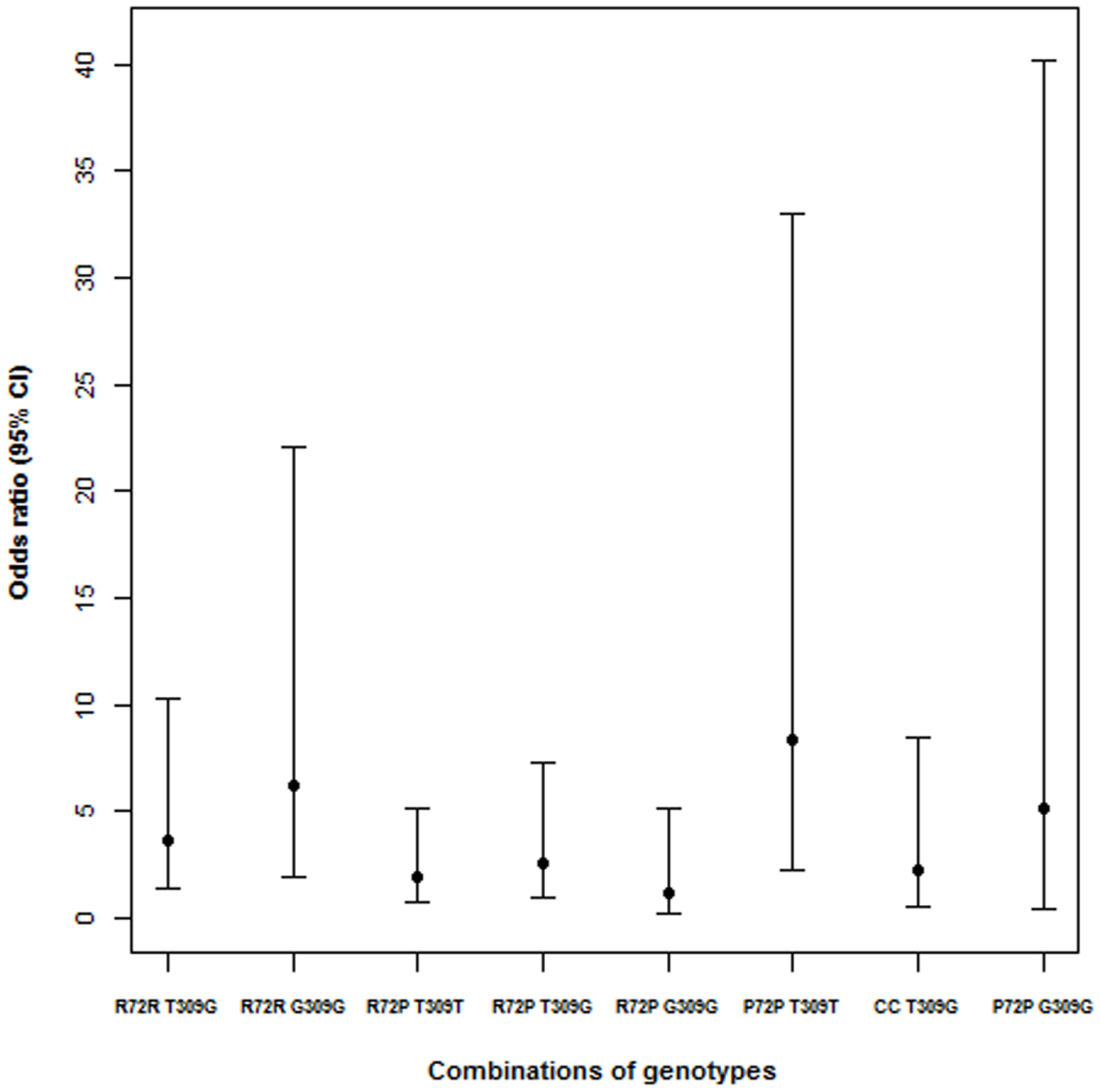
OR (with 95% CI) for the association (adjusted for skin color and age) between combined genotypes of the two SNPs and HIV status.

Since there were significant associations of different epistaticmodels with HIV status, a visual inspection of OR (with 95% CI) resulting from the adjusted analyses between genotypic combinations of the two SNPs and HIV was performed to identify patterns and, possibly, elaborate further epistatic models. Larger (and similar to one another) OR values were observed for the genotypic combinations R72R G309G and P72PT309T than for the rest (figure 1). This pattern indicates thata different epistatic effect may be underlying the observed associations. Therefore, an additional model was elaborated to reflect the following epistatic relationship: a double dominant effect that both affects disease risk and blocks a “hidden” additional recessive effect, which is manifested in the presence of a homozygous-wild homozygous-variant genotypic combination (which could be called double dominant epistasis with blocked recessive effects). Such model was tested in categorical [assuming distinct effects for the following genotypic combinations: R72R T309T (reference); R72P _309G_, _72_ T309G and P72P G309G; and R72R G309G and P72P T309T] and in numeric (i.e., linear tendency) forms, hereafter referred to as models 9.1 and 9.2, respectively. Even stronger associations than the previous ones were observed for models 9.1 [genotypic combinations R72P _309G_ - _72_ T309G - P72P G309G and R72R G309G - P72P T309T, respectively; crude: OR (95% CI), 2.43 (1.15-5.77) and 6.87 (2.72-18.91); P<0.001; adjusted: OR (95% CI), 2.47 (1.12-6.06) and 7.08 (2.59-21.01); P = 0.001] and 9.2 (crude: OR (95% CI), 2.66 (1.67-4.35); P<0.001; adjusted: OR (95% CI), 2.68 (1.62-4.60); P<0.001].

## Discussion

We investigated,for the first time in the epidemiological setting, the evidence linking p53 and MDM2 with HIV,as well aspotential implications of SNPs in the p53 pathway for HPV-related outcomes others than cancer development and/or progression. Analyses of epistatic models provided evidence for a novel mechanism linking the p53 pathway with HIV infection status. Interestingly, we observed large effect sizes for the epistatic models (achieving notably low P-values in a relatively small sample). This contrasts with a recent GWAS involving 6300 cases and 7200 controls that tested approximately 8 million common variants, which suggested that host genetic influences on HIV acquisition are either rare or have very small effects (notdetectable given the study power) [20]. In our study, all genotypic combinations of the epistatic model 9.1 had a relatively high prevalence [R72R T309T: 50 (20.0%); R72R G309G - P72P T309T: 20 (8.0%) and the remaining genotypic combinations: 180 (72.0%) in HIV-positive and R72R T309T: 8 (8.0%); R72R G309G - P72P T309T: 22 (22.0%) and the remaininggenotypic combinations: 70 (70.0%) in HIV-negative women] and large effect sizes (OR, others: 2.47; R72R G309G - P72P T309T: 7.08). Indeed, when comparing our findings with the cited GWAS studies on HIV acquisition[15], [20], our effect sizes are larger than all of the OR of statistically-significant SNPs reported in these studies, with one exception (OR, 9.51) thatwas observed in an exploratory phase of the analyses but was markedly decreased in the expanded and external validation analyses (2.67 and 1.69, respectively) [18]. This observation reinforces the importance of analyzing epistasis even in genome-wide scalestudies [47], [48].

An important consideration is that we found relatively weak associations of epistatic models with HPV-related outcomes (especially for HPV status), which would not be expected given their strong associations with HIV status. While very strong associations were found between some epistaticmodels and HIV status, there were no associations of models 9.1 and 9.2 with HPV status and HPV oncogenic risk (data not shown). It is conceptually difficult to conceive a factor causally involved in HIV status but not with HPV status when this is not adjusted for in the analysis (unless HIV status does not have a causal effect on HPV status). However, this issue does not invalidate our findings, since they are unlikely to be caused by confounding (discussed below) or chance alone (the P-values for models 9.1 and 9.2 were very low).

An important limitation of our study was the sample size (which increase was not possible due to practical/logistic reasons). Power analysesshowed that, as expected, there was generally low power (power <0.80) when HPV oncogenic risk was the dependent variable (power <0.80) for single-SNP and epistasis analyses (due to reduced sample size) alike. For the former (table S6), associations involving OR<2 and performed under the recessive model (of which the exposure prevalence is smaller than of other genetic effects) were also generally underpowered. For the latter (table S7), power was also reduced for associations involving OR<3 and for several models when HIV status was the dependent variable. These results, in addition to the partial redundancy of some genetic/epistatic models, are reflected in the several marginal associations observed. Although these observationsillustrate that our analyses were underpowered under some circumstances, they support the causality of our associations, since the effect sizes were large enough to achieve statistical significance even in a small sample. This is particularly illustrated in the epistatic model 9.1: while power analyses indicate low power when HIV status was the dependent variable, very strong associations were observed due to the large OR values.

Another important consideration is thatthe study design was not optimal for causal inference. This is particularly relevant considering the hypothesis that the SNPs (in combination) might influence virus establishment/persistence after exposure to it, which would be a rather dynamic mechanism. However, it is well-known that associations involving germ-line genetic markers as independent variables are not subjected to reverse causation and are generally robust against confounding, as reviewed in the context of Mendelian randomization [49]. To further reduce the possibility of residual confounding caused by population stratification, the analyses were adjusted for skin color and additional covariates. Nonetheless, the optimal design would be a prospective study to validate our findings and understand their underlying mechanisms. Besides, the results for models 9.1 and 9.2 were very similar, thus requiring additional studies for validating not only the associations, but also the actual model epistaticmodel. Another issue is the true relationship among genetic markers and the study outcomes: it cannot be determined from our study whether the SNPs are truly causal or are in linkage disequilibrium with the causal variants. Nevertheless, the value of our findings regarding genetic predisposition to HIV persistence after viral exposure is independent of this issue.In summary, ourresults provided evidence for a role of the p53 pathway – involving R72P and T309G SNPs – in HPV status and HPV oncogenic risk, and strong associations were found for an epistatic effect on HIV status. Our results require validation in prospective cohort studies using larger samples for the associations and to identify the best epistatic model. Applications of our findings are related to genetic testing for HIV susceptibility and contributing to the understanding of HIV susceptibility differences among populations. Furthermore, findings of this nature can be explored in laboratory studies aiming at either elucidating HIV pathogenesis or developing new therapeutic/preventive strategies.

## Supporting Information

Table S1 **Likelihood-ratio chi-squared tests P-values of the selection of confounders based on association with each outcome.** ^*^Skin color was included as a covariate regardless of meeting the selection criteria.
(DOCX)

Table S2 **Likelihood-ratio chi-squared tests P-values of the selection of confounders based on association with each SNP.** ^*^Skin color was included as a covariate regardless of meeting the selection criteria. ^†^Also associated with HPV and HIV status.
(DOCX)

Table S3 **Adjusted associations [showing OR (95% CI) and P-values], including HIV status as a covariate, between R72P SNP and HPV status and between T309G SNP and HPV oncogenic risk.** ^*^“A” and “a” correspond to wild-type (i.e., either R72 or T309) and variant alleles (i.e., either P72 or G309), respectively.
(DOCX)

Table S4 **Likelihood-ratio chi-squared tests P-values of the selection of confounders based on association with combined genotypes.** ^*^Skin color was included as a covariate regardless of meeting the selection criteria. ^†^Also associated with HPV and HIV status.
(DOCX)

Table S5 **Adjusted associations (including HIV status as a covariate) between 11 epistatic models and HPV oncogenic risk.** ^*^The epistatic models were numbered as described previously [46]. ^†^The “_” indicates that the effect is irrespective of the allele. E.g., R72P G309_ represents the genotypic combinations R72P T309G - R72P G309G. ^¥^Other: **3.1**: _72_ T309_ - R72R _309_. **3.2**: R72_ _309_ - _72_ T309T. **4**: P72_ _309_ - _72_ G309_. **5**: P72P _309_ - _72_ G309G. **6**: R72R G309_ - P72_ T309T. **7**: P72P T309_ - R72_ G309G.
(DOCX)

Table S6 **Statistical power of the single-SNP analyses for different OR values.** ^*^The OR values of 1.25, 1.5, 2.0 and 3.0 correspond to 0.80, 0.67, 0.50 and 0.33, respectively.
(DOCX)

Table S7 **Statistical power of the epistasis analyses for different OR values.** ^*^The OR values of 1.25, 1.5, 2.0 and 3.0 correspond to 0.80, 0.67, 0.50 and 0.33, respectively.
(DOCX)

Methods S1 **Power Analysis.**
(DOCX)
