# Supplementary material for: Evidence for an Epistatic Effect between TP53 R72P and MDM2 T309G SNPs in HIV Infection: A Cross-Sectional Study in Women from South Brazil
Source: PLoS One. 2014 Feb 28;9(2):e89489. doi: 10.1371/journal.pone.0089489 (PMC3938491; doi:10.1371/journal.pone.0089489)
Supplement: Table S1 — Likelihood-ratio chi-squared tests P-values of the selection of confounders based on association with each outcome. *Skin color was included as a covariate regardless of meeting the selection criteria. (DOCX) [file pone.0089489.s001.docx]

| **Variables** | **HPV status** | **HPV oncogenic risk** | | | **HIV status** | |
| --- | --- | --- | --- | --- | --- | --- |
|  | **Step 1** | **Step 1** | **Step 2** | **Step 3** | **Step 1** | **Step 2** |
| **Skin color^*^** | 0.722 | 0.498 | 0.799 | 0.753 | 0.061 | 0.002 |
| **Age** | 0.002 | 0.513 | 0.335 | - | <0.001 | <0.001 |
| **Schooling** | 0.084 | 0.313 | 0.232 | 0.043 | 0.002 | <0.001 |
| **Family income** | 0.007 | 0.824 | - | - | 0.255 | - |
