# Supplementary material for: Evidence for an Epistatic Effect between TP53 R72P and MDM2 T309G SNPs in HIV Infection: A Cross-Sectional Study in Women from South Brazil
Source: PLoS One. 2014 Feb 28;9(2):e89489. doi: 10.1371/journal.pone.0089489 (PMC3938491; doi:10.1371/journal.pone.0089489)
Supplement: Table S2 — Likelihood-ratio chi-squared tests P-values of the selection of confounders based on association with each SNP. *Skin color was included as a covariate regardless of meeting the selection criteria. †Also associated with HPV and HIV status. (DOCX) [file pone.0089489.s002.docx]

| **Variables** | **TP53** | | | **MDM2** | | |
| --- | --- | --- | --- | --- | --- | --- |
|  | **Step 1** | **Step 2** | **Step 3** | **Step 1** | **Step 2** | **Step 3** |
| **Skin color^*^** | 0.026 | 0.029 | 0.003 | <0.001 | 0.001 | 0.001 |
| **Age** | 0.913 | - | - | 0.145 | 0.117 | 0.194^†^ |
| **Schooling** | 0.350 | 0.365 | 0.384 | 0.908 | 0.787 | - |
| **Family income** | 0.500 | 0.453 | - | 0.937 | - | - |

**Table S3.** Adjusted associations [showing OR (95% CI) and P-values], including HIV status as a covariate, between R72P SNP and HPV status and between T309G SNP and HPV oncogenic risk.

| **Genetic model^*^** | **R72P** | **T309G** |
| --- | --- | --- |
| **Codominant** | P=0.100 | P=0.109 |
| A/A | 1 (Reference) | 1 (Reference) |
| A/a | 0.60 (0.36-0.99) | 0.43 (0.15-1.21) |
| a/a | 1.00 (0.48-2.10) | 1.44 (0.34-6.61) |
| **Overdominant** | P=0.032 | P=0.040 |
| A/A-a/a | 1 (Reference) | 1 (Reference) |
| A/a | 0.60 (0.38-0.96) | 0.38 (0.14-0.96) |
| **Additive** | P=0.420 | P=0.953 |
| N^o^ of “a” alleles | 0.87 (0.61-1.23) | 0.98 (0.49-1.96) |
| **Dominant** | P=0.099 | P=0.255 |
| A/A | 1 (Reference) | 1 (Reference) |
| A/a-a/a | 0.67 (0.42-1.08) | 0.56 (0.20-1.51) |
| **Recessive** | P=0.422 | P=0.172 |
| A/A-A/a | 1 (Reference) | 1 (Reference) |
| a/a | 1.33 (0.66-2.63) | 2.46 (0.68-9.87) |

^*^”A” and “a” correspond to wild-type (i.e., either R72 or T309) and variant alleles (i.e., either P72 or G309), respectively.

**Table S4.** Likelihood-ratio chi-squared tests P-values of the selection of confounders based on association with combined genotypes.

| **Variables** | **Step 1** | **Step 2** | **Step 3** |
| --- | --- | --- | --- |
| **Skin color^*^** | <0.001 | <0.001 | <0.001 |
| **Age** | 0.455 | 0.352 | 0.164^†^ |
| **Schooling** | 0.867 | - | - |
| **Family income** | 0.855 | 0.846 | - |

^*^Skin color was included as a covariate regardless of meeting the selection criteria. ^†^Also associated with HPV and HIV status.

**Table S5.** Adjusted associations (including HIV status as a covariate) between 11 epistatic models and HPV oncogenic risk.

| **Epistatic model^*^** | **Genotypic**  **Combination** | **OR (95% CI)** | **P-value** |
| --- | --- | --- | --- |
|  |  |  |  |
| Dominant (1.1) | R72R T309T | 1 (Reference) | P=0.594 |
|  | R72P G309_^†^ | 0.60 (0.12-2.75) |  |
|  | P72_ _309_ | 1.02 (0.21-4.44) |  |
| Dominant (1.2) | R72R T309T | 1 (Reference) | P=0.482 |
|  | P72_ T309T | 1.43 (0.24-8.16) |  |
|  | _72_ G309_ | 0.70 (0.15-2.89) |  |
| Recessive (2.1) | R72_ T309_ | 1 (Reference) | P=0.236 |
|  | R72_ G309G | 3.32 (0.81-15.64) |  |
|  | P72P _309_ | 1.45 (0.37-6.09) |  |
| Recessive (2.2) | R72_ T309_ | 1 (Reference) | P=0.306 |
|  | R72R T309_ | 1.70 (0.40-7.78) |  |
|  | _72_ G309G | 2.63 (0.71-10.71) |  |
| Dominant and  recessive (3.1) | Other^¥^ | 1 (Reference) | P=0.282 |
|  | P72_ G309G | 3.36 (0.41-73.33) |  |
| Dominant and  recessive (3.2) | Other | 1 (Reference) | P=0.957 |
|  | P72P G309_ | 1.05 (0.18-6.70) |  |
| Double dominant (no effect accumulation) (4) | R72R T309T | 1 (Reference) | P=0.775 |
|  | Other | 0.81 (0.18-3.30) |  |
| Double recessive (no effect accumulation) (5) | R72_ T309_ | 1 (Reference) | P=0.142 |
|  | Other | 2.17 (0.77-6.47) |  |
| Double dominant (with effect accumulation) (6) | R72R T309T | 1 (Reference) | P=0.957 |
|  | Other | 0.82 (0.17-3.54) |  |
|  | P72_ G309_ | 0.79 (0.15-3.75) |  |
| Double recessive (with effect accumulation) (7) | R72_ T309_ | 1 (Reference) | P=0.273 |
|  | Other | 2.37 (0.82-7.29) |  |
|  | P72P G309G | 0.79 (0.04-26.33) |  |
| Quantitative (8) | N^o^ of P72 and G309 alleles | 1.14 (0.69-1.95) | P=0.611 |
|  |  |  |  |

^*^The epistatic models were numbered as described previously [46].

^†^The “_” indicates that the effect is irrespective of the allele. E.g., R72P G309_ represents the genotypic combinations R72P T309G - R72P G309G.

^¥^Other: **3.1:** _72_ T309_ - R72R _309_. **3.2:** R72_ _309_ - _72_ T309T. **4:** P72_ _309_ - _72_ G309_. **5:** P72P _309_ - _72_ G309G. **6:** R72R G309_ - P72_ T309T. **7:** P72P T309_ - R72_ G309G.

**Table S6.** Statistical power of the single-SNP analyses for different OR values.

| **OR^*^** | **Genetic** | **HPV status** | | **HPV oncogenic risk** | | **HIV status** | |
| --- | --- | --- | --- | --- | --- | --- | --- |
|  | **Effect** | **SNPs** | | **SNPs** | | **SNPs** | |
|  |  | **R72P** | **T309G** | **R72P** | **T309G** | **R72P** | **T309G** |
| 1.25 | Codominant | 0.163 | 0.163 | 0.092 | 0.079 | 0.128 | 0.108 |
|  | Overdominant | 0.185 | 0.170 | 0.090 | 0.077 | 0.150 | 0.118 |
|  | Additive | 0.291 | 0.306 | 0.134 | 0.116 | 0.232 | 0.194 |
|  | Dominant | 0.176 | 0.172 | 0.098 | 0.079 | 0.148 | 0.116 |
|  | Recessive | 0.106 | 0.110 | 0.077 | 0.072 | 0.102 | 0.092 |
| 1.5 | Codominant | 0.424 | 0.408 | 0.164 | 0.131 | 0.346 | 0.241 |
|  | Overdominant | 0.471 | 0.459 | 0.183 | 0.143 | 0.400 | 0.305 |
|  | Additive | 0.726 | 0.761 | 0.301 | 0.251 | 0.646 | 0.546 |
|  | Dominant | 0.466 | 0.455 | 0.190 | 0.139 | 0.386 | 0.270 |
|  | Recessive | 0.252 | 0.269 | 0.117 | 0.120 | 0.210 | 0.183 |
| 2.0 | Codominant | 0.842 | 0.846 | 0.374 | 0.258 | 0.787 | 0.625 |
|  | Overdominant | 0.890 | 0.886 | 0.408 | 0.307 | 0.859 | 0.726 |
|  | Additive | 0.989 | 0.995 | 0.640 | 0.533 | 0.983 | 0.964 |
|  | Dominant | 0.882 | 0.893 | 0.426 | 0.292 | 0.836 | 0.692 |
|  | Recessive | 0.580 | 0.630 | 0.218 | 0.244 | 0.512 | 0.474 |
| 3.0 | Codominant | 0.997 | 0.998 | 0.702 | 0.502 | 0.995 | 0.969 |
|  | Overdominant | 0.999 | 0.999 | 0.732 | 0.568 | 0.998 | 0.992 |
|  | Additive | 0.999 | 0.999 | 0.914 | 0.815 | 0.999 | 0.999 |
|  | Dominant | 0.998 | 0.999 | 0.764 | 0.546 | 0.998 | 0.984 |
|  | Recessive | 0.917 | 0.944 | 0.410 | 0.449 | 0.893 | 0.879 |

^*^The OR values of 1.25, 1.5, 2.0 and 3.0 correspond to 0.80, 0.67, 0.50 and 0.33, respectively.

**Table S7.** Statistical power of the epistasis analyses for different OR values.

| **Epistatic model** | **HPV status** | | | | **HPV oncogenic risk** | | | | **HIV status** | | | |
| --- | --- | --- | --- | --- | --- | --- | --- | --- | --- | --- | --- | --- |
|  | **OR^*^** | | | | **OR^*^** | | | | **OR^*^** | | | |
|  | **1.25** | **1.5** | **2.0** | **3.0** | **1.25** | **1.5** | **2.0** | **3.0** | **1.25** | **1.5** | **2.0** | **3.0** |
| 1.1 | 0.103 | 0.221 | 0.534 | 0.884 | 0.072 | 0.092 | 0.152 | 0.263 | 0.076 | 0.111 | 0.229 | 0.558 |
| 1.2 | 0.103 | 0.229 | 0.544 | 0.898 | 0.074 | 0.094 | 0.152 | 0.250 | 0.068 | 0.110 | 0.243 | 0.571 |
| 2.1 | 0.110 | 0.236 | 0.572 | 0.917 | 0.077 | 0.109 | 0.210 | 0.404 | 0.087 | 0.173 | 0.419 | 0.829 |
| 2.2 | 0.100 | 0.218 | 0.521 | 0.877 | 0.071 | 0.109 | 0.195 | 0.364 | 0.084 | 0.156 | 0.382 | 0.776 |
| 3.1 | 0.096 | 0.188 | 0.424 | 0.794 | 0.082 | 0.104 | 0.166 | 0.295 | 0.076 | 0.147 | 0.346 | 0.721 |
| 3.2 | 0.082 | 0.161 | 0.378 | 0.740 | 0.082 | 0.112 | 0.173 | 0.288 | 0.077 | 0.130 | 0.303 | 0.647 |
| 4 | 0.123 | 0.287 | 0.665 | 0.963 | 0.078 | 0.103 | 0.181 | 0.323 | 0.077 | 0.125 | 0.308 | 0.710 |
| 5 | 0.150 | 0.374 | 0.806 | 0.994 | 0.081 | 0.156 | 0.328 | 0.622 | 0.108 | 0.249 | 0.622 | 0.964 |
| 6 | 0.115 | 0.262 | 0.627 | 0.944 | 0.073 | 0.101 | 0.172 | 0.303 | 0.075 | 0.126 | 0.279 | 0.667 |
| 7 | 0.145 | 0.374 | 0.782 | 0.991 | 0.086 | 0.151 | 0.316 | 0.599 | 0.107 | 0.246 | 0.608 | 0.953 |
| 8 | 0.514 | 0.945 | 0.999 | 0.999 | 0.176 | 0.414 | 0.726 | 0.920 | 0.322 | 0.846 | 0.999 | 0.999 |
| 9.1 | 0.123 | 0.284 | 0.654 | 0.958 | 0.072 | 0.105 | 0.181 | 0.315 | 0.076 | 0.133 | 0.309 | 0.689 |
| 9.2 | 0.194 | 0.515 | 0.921 | 0.999 | 0.090 | 0.166 | 0.328 | 0.556 | 0.092 | 0.237 | 0.638 | 0.987 |

^*^The OR values of 1.25, 1.5, 2.0 and 3.0 correspond to 0.80, 0.67, 0.50 and 0.33, respectively.
