# Supplementary material for: Evidence for an Epistatic Effect between TP53 R72P and MDM2 T309G SNPs in HIV Infection: A Cross-Sectional Study in Women from South Brazil
Source: PLoS One. 2014 Feb 28;9(2):e89489. doi: 10.1371/journal.pone.0089489 (PMC3938491; doi:10.1371/journal.pone.0089489)
Supplement: Table S3 — Adjusted associations [showing OR (95% CI) and P-values], including HIV status as a covariate, between R72P SNP and HPV status and between T309G SNP and HPV oncogenic risk. *“A” and “a” correspond to wild-type (i.e., either R72 or T309) and variant alleles (i.e., either P72 or G309), respectively. (DOCX) [file pone.0089489.s003.docx]

| **Genetic model^*^** | **R72P** | **T309G** |
| --- | --- | --- |
| **Codominant** | P=0.100 | P=0.109 |
| A/A | 1 (Reference) | 1 (Reference) |
| A/a | 0.60 (0.36-0.99) | 0.43 (0.15-1.21) |
| a/a | 1.00 (0.48-2.10) | 1.44 (0.34-6.61) |
| **Overdominant** | P=0.032 | P=0.040 |
| A/A-a/a | 1 (Reference) | 1 (Reference) |
| A/a | 0.60 (0.38-0.96) | 0.38 (0.14-0.96) |
| **Additive** | P=0.420 | P=0.953 |
| N^o^ of “a” alleles | 0.87 (0.61-1.23) | 0.98 (0.49-1.96) |
| **Dominant** | P=0.099 | P=0.255 |
| A/A | 1 (Reference) | 1 (Reference) |
| A/a-a/a | 0.67 (0.42-1.08) | 0.56 (0.20-1.51) |
| **Recessive** | P=0.422 | P=0.172 |
| A/A-A/a | 1 (Reference) | 1 (Reference) |
| a/a | 1.33 (0.66-2.63) | 2.46 (0.68-9.87) |
