# Supplementary material for: Evidence for an Epistatic Effect between TP53 R72P and MDM2 T309G SNPs in HIV Infection: A Cross-Sectional Study in Women from South Brazil
Source: PLoS One. 2014 Feb 28;9(2):e89489. doi: 10.1371/journal.pone.0089489 (PMC3938491; doi:10.1371/journal.pone.0089489)
Supplement: Table S4 — Likelihood-ratio chi-squared tests P-values of the selection of confounders based on association with combined genotypes. *Skin color was included as a covariate regardless of meeting the selection criteria. †Also associated with HPV and HIV status. (DOCX) [file pone.0089489.s004.docx]

| **Variables** | **Step 1** | **Step 2** | **Step 3** |
| --- | --- | --- | --- |
| **Skin color^*^** | <0.001 | <0.001 | <0.001 |
| **Age** | 0.455 | 0.352 | 0.164^†^ |
| **Schooling** | 0.867 | - | - |
| **Family income** | 0.855 | 0.846 | - |
