# Supplementary material for: Evidence for an Epistatic Effect between TP53 R72P and MDM2 T309G SNPs in HIV Infection: A Cross-Sectional Study in Women from South Brazil
Source: PLoS One. 2014 Feb 28;9(2):e89489. doi: 10.1371/journal.pone.0089489 (PMC3938491; doi:10.1371/journal.pone.0089489)
Supplement: Table S5 — Adjusted associations (including HIV status as a covariate) between 11 epistatic models and HPV oncogenic risk. *The epistatic models were numbered as described previously [46]. †The “_” indicates that the effect is irrespective of the allele. E.g., R72P G309_ represents the genotypic combinations R72P T309G - R72P G309G. ¥Other: 3.1: _72_ T309_ - R72R _309_. 3.2: R72_ _309_ - _72_ T309T. 4: P72_ _309_ - _72_ G309_. 5: P72P _309_ - _72_ G309G. 6: R72R G309_ - P72_ T309T. 7: P72P T309_ - R72_ G309G. (DOCX) [file pone.0089489.s005.docx]

| **Epistaticmodel^*^** | **Genotypic** | **OR (95% CI)** | **P-value** |
| --- | --- | --- | --- |
|  | **combination** |  |  |
| Dominant (1.1) | R72R T309T | 1 (Reference) | P=0.594 |
|  | R72R G309_^†^ | 0.60 (0.12-2.75) |  |
|  | P72_ _309_ | 1.02 (0.21-4.44) |  |
| Dominant (1.2) | R72R T309T | 1 (Reference) | P=0.482 |
|  | P72_ T309T | 1.43 (0.24-8.16) |  |
|  | _72_ G309_ | 0.70 (0.15-2.89) |  |
| Recessive (2.1) | R72_ T309_ | 1 (Reference) | P=0.236 |
|  | R72_ G309G | 3.32 (0.81-15.64) |  |
|  | P72P _309_ | 1.45 (0.37-6.09) |  |
| Recessive(2.2) | R72_ T309_ | 1 (Reference) | P=0.306 |
|  | R72R T309_ | 1.70 (0.40-7.78) |  |
|  | _72_ G309G | 2.63 (0.71-10.71) |  |
| Dominantand | Other^¥^ | 1 (Reference) | P=0.282 |
| recessive (3.1) | P72_ G309G | 3.36 (0.41-73.33) |  |
| Dominantand | Other | 1 (Reference) | P=0.957 |
| recessive (3.2) | P72PG309_ | 1.05 (0.18-6.70) |  |
| Double dominant(no | R72R T309T | 1 (Reference) | P=0.775 |
| effect accumulation) (4) | Other | 0.81 (0.18-3.30) |  |
| Double recessive (no | R72_ T309_ | 1 (Reference) | P=0.142 |
| effect accumulation) (5) | Other | 2.17 (0.77-6.47) |  |
| Double dominant(with | R72R T309T | 1 (Reference) | P=0.957 |
| effect accumulation) (6) | Other | 0.82 (0.17-3.54) |  |
|  | P72_ G309_ | 0.79 (0.15-3.75) |  |
| Double recessive (with | R72_ T309_ | 1 (Reference) | P=0.273 |
| effect accumulation) (7) | Other | 2.37 (0.82-7.29) |  |
|  | P72P G309G | 0.79 (0.04-26.33) |  |
| Quantitative (8) | N^o^ of P72 and | 1.14 (0.69-1.95) | P=0.611 |
|  | G309 alleles |  |  |
