# Supplementary material for: Evidence for an Epistatic Effect between TP53 R72P and MDM2 T309G SNPs in HIV Infection: A Cross-Sectional Study in Women from South Brazil
Source: PLoS One. 2014 Feb 28;9(2):e89489. doi: 10.1371/journal.pone.0089489 (PMC3938491; doi:10.1371/journal.pone.0089489)
Supplement: Table S6 — Statistical power of the single-SNP analyses for different OR values. *The OR values of 1.25, 1.5, 2.0 and 3.0 correspond to 0.80, 0.67, 0.50 and 0.33, respectively. (DOCX) [file pone.0089489.s006.docx]

| **OR^*^** | **Genetic** | **HPV status** | | **HPV oncogenic risk** | | **HIV status** | |
| --- | --- | --- | --- | --- | --- | --- | --- |
|  | **Effect** | **SNPs** | | **SNPs** | | **SNPs** | |
|  |  | **R72P** | **T309G** | **R72P** | **T309G** | **R72P** | **T309G** |
| 1.25 | Codominant | 0.163 | 0.163 | 0.092 | 0.079 | 0.128 | 0.108 |
|  | Overdominant | 0.185 | 0.170 | 0.090 | 0.077 | 0.150 | 0.118 |
|  | Additive | 0.291 | 0.306 | 0.134 | 0.116 | 0.232 | 0.194 |
|  | Dominant | 0.176 | 0.172 | 0.098 | 0.079 | 0.148 | 0.116 |
|  | Recessive | 0.106 | 0.110 | 0.077 | 0.072 | 0.102 | 0.092 |
| 1.5 | Codominant | 0.424 | 0.408 | 0.164 | 0.131 | 0.346 | 0.241 |
|  | Overdominant | 0.471 | 0.459 | 0.183 | 0.143 | 0.400 | 0.305 |
|  | Additive | 0.726 | 0.761 | 0.301 | 0.251 | 0.646 | 0.546 |
|  | Dominant | 0.466 | 0.455 | 0.190 | 0.139 | 0.386 | 0.270 |
|  | Recessive | 0.252 | 0.269 | 0.117 | 0.120 | 0.210 | 0.183 |
| 2.0 | Codominant | 0.842 | 0.846 | 0.374 | 0.258 | 0.787 | 0.625 |
|  | Overdominant | 0.890 | 0.886 | 0.408 | 0.307 | 0.859 | 0.726 |
|  | Additive | 0.989 | 0.995 | 0.640 | 0.533 | 0.983 | 0.964 |
|  | Dominant | 0.882 | 0.893 | 0.426 | 0.292 | 0.836 | 0.692 |
|  | Recessive | 0.580 | 0.630 | 0.218 | 0.244 | 0.512 | 0.474 |
| 3.0 | Codominant | 0.997 | 0.998 | 0.702 | 0.502 | 0.995 | 0.969 |
|  | Overdominant | 0.999 | 0.999 | 0.732 | 0.568 | 0.998 | 0.992 |
|  | Additive | 0.999 | 0.999 | 0.914 | 0.815 | 0.999 | 0.999 |
|  | Dominant | 0.998 | 0.999 | 0.764 | 0.546 | 0.998 | 0.984 |
|  | Recessive | 0.917 | 0.944 | 0.410 | 0.449 | 0.893 | 0.879 |
