# Supplementary material for: Evidence for an Epistatic Effect between TP53 R72P and MDM2 T309G SNPs in HIV Infection: A Cross-Sectional Study in Women from South Brazil
Source: PLoS One. 2014 Feb 28;9(2):e89489. doi: 10.1371/journal.pone.0089489 (PMC3938491; doi:10.1371/journal.pone.0089489)
Supplement: Table S7 — Statistical power of the epistasis analyses for different OR values. *The OR values of 1.25, 1.5, 2.0 and 3.0 correspond to 0.80, 0.67, 0.50 and 0.33, respectively. (DOCX) [file pone.0089489.s007.docx]

| **Epistatic model** | **HPV status** | | | | **HPV oncogenic risk** | | | | **HIV status** | | | |
| --- | --- | --- | --- | --- | --- | --- | --- | --- | --- | --- | --- | --- |
|  | **OR^*^** | | | | **OR^*^** | | | | **OR^*^** | | | |
|  | **1.25** | **1.5** | **2.0** | **3.0** | **1.25** | **1.5** | **2.0** | **3.0** | **1.25** | **1.5** | **2.0** | **3.0** |
| 1.1 | 0.103 | 0.221 | 0.534 | 0.884 | 0.072 | 0.092 | 0.152 | 0.263 | 0.076 | 0.111 | 0.229 | 0.558 |
| 1.2 | 0.103 | 0.229 | 0.544 | 0.898 | 0.074 | 0.094 | 0.152 | 0.250 | 0.068 | 0.110 | 0.243 | 0.571 |
| 2.1 | 0.110 | 0.236 | 0.572 | 0.917 | 0.077 | 0.109 | 0.210 | 0.404 | 0.087 | 0.173 | 0.419 | 0.829 |
| 2.2 | 0.100 | 0.218 | 0.521 | 0.877 | 0.071 | 0.109 | 0.195 | 0.364 | 0.084 | 0.156 | 0.382 | 0.776 |
| 3.1 | 0.096 | 0.188 | 0.424 | 0.794 | 0.082 | 0.104 | 0.166 | 0.295 | 0.076 | 0.147 | 0.346 | 0.721 |
| 3.2 | 0.082 | 0.161 | 0.378 | 0.740 | 0.082 | 0.112 | 0.173 | 0.288 | 0.077 | 0.130 | 0.303 | 0.647 |
| 4 | 0.123 | 0.287 | 0.665 | 0.963 | 0.078 | 0.103 | 0.181 | 0.323 | 0.077 | 0.125 | 0.308 | 0.710 |
| 5 | 0.150 | 0.374 | 0.806 | 0.994 | 0.081 | 0.156 | 0.328 | 0.622 | 0.108 | 0.249 | 0.622 | 0.964 |
| 6 | 0.115 | 0.262 | 0.627 | 0.944 | 0.073 | 0.101 | 0.172 | 0.303 | 0.075 | 0.126 | 0.279 | 0.667 |
| 7 | 0.145 | 0.374 | 0.782 | 0.991 | 0.086 | 0.151 | 0.316 | 0.599 | 0.107 | 0.246 | 0.608 | 0.953 |
| 8 | 0.514 | 0.945 | 0.999 | 0.999 | 0.176 | 0.414 | 0.726 | 0.920 | 0.322 | 0.846 | 0.999 | 0.999 |
| 9.1 | 0.123 | 0.284 | 0.654 | 0.958 | 0.072 | 0.105 | 0.181 | 0.315 | 0.076 | 0.133 | 0.309 | 0.689 |
| 9.2 | 0.194 | 0.515 | 0.921 | 0.999 | 0.090 | 0.166 | 0.328 | 0.556 | 0.092 | 0.237 | 0.638 | 0.987 |
