# Supplementary material for: Evidence for an Epistatic Effect between TP53 R72P and MDM2 T309G SNPs in HIV Infection: A Cross-Sectional Study in Women from South Brazil
Source: PLoS One. 2014 Feb 28;9(2):e89489. doi: 10.1371/journal.pone.0089489 (PMC3938491; doi:10.1371/journal.pone.0089489)
Supplement: Methods S1 — Power Analysis. (DOCX) [file pone.0089489.s008.docx]

**Methods S1**

**Power Analysis**

Given our limited sample size and the practical/logistic impossibility of increasing it, power analyses were performed to estimate the statistical power of this study for different OR values. For each association, power analysis was performed by 10000 simulations. Each simulated dataset was created using a weighted (according to the prevalence of each exposure observed in the actual data) sample of exposure status (of size equal to the sample size) and a random sample from the binomial distribution (of size equal to the sample size and the probability of success of each trial defined as $\frac{Odds of disease among exposed individuals}{1+ Odds of disease among exposed individuals}$). The parameters were: sample size (350 for HPV status and HIV status, and 126 for HPV oncogenic risk; Table 1), OR for the exposure (1.25, 1.5, 2.0, and 3.0, which are equivalent, regarding power analysis, to 0.80, 0.67, 0.50 and 0.33, respectively), odds of disease among the unexposed (defined as the exponential of the intercept of the respective adjusted analyses) and prevalence of exposure (defined according to the actual data). For the models codominant, 1.1., 1.2, 2.1, 2.2, 6, 7 and 9.1, each genotype or genotypic combination was assumed to have the same effect on the outcome. Each simulated dataset was analyzed by logistic regression, and P-values were obtained using likelihood-ratio chi-squared test. The estimated power of a given association was defined as the proportion of simulations that yielded a P-value < 0.05.
